# *De novo* transcriptome dataset of a *Mayorella* species isolated from deep sea

**DOI:** 10.1016/j.dib.2025.111864

**Published:** 2025-07-09

**Authors:** Wenli Guo, Xiaoli Lei, Chen Liang, Jianming Chen

**Affiliations:** a College of Oceanography, Fujian Agriculture and Forestry University, 350002 Fuzhou, Fujian, PR China; b Fujian Key Laboratory on Conservation and Sustainable Utilization of Marine Biodiversity, Fuzhou Institute of Oceanography, College of Geography and Oceanography, Minjiang University, 350108 Fuzhou, PR China; c Technology Innovation Center for Monitoring and Restoration Engineering of Ecological Fragile Zone in Southeast China, Ministry of Natural Resources, 350001 Fujian, PR China

**Keywords:** Amoebozoa, RNA, Functional annotation, Mariana trench area

## Abstract

*Mayorella marianaensis* (Amoebozoa: Discosea) was the only *Mayorella* species isolated from deep sea (over 3000 m-depth). We firstly present a transcriptomic analysis of the non-model amoeba species collected from the Mariana Trench area. Illumina sequencing platform was used to generate data which including raw data, cleaned reads, *de novo* assembly, and functional annotation. After assembly, the final transcriptome consists of 57,459,544 transcripts with a mean length of 1646 and N50 length of 1170. The transcriptome has a completeness of 67.4 % as assessed by BUSCO. Functional annotation pathways related to signal transduction, transport and catabolism, and translation are the most annotated in the transcriptome.

Specifications table

**Table:** 

Subject	Omics: Transcriptomics
Specific subject area	Marine amoeba transcriptomics.
Type of data	Table, Figure, Raw, Assembly
Data collection	*Mayorella marianaensis* total RNA was extracted using Trizol, and analysed with the Agilent 2100 Bioanalyzer. Libraries were sequenced in the Illumina HiSeq platform.
Data source location	Institution: Minjiang University City/Town/Region: Shangjie Town, Minhou County, Fuzhou City Country: China Deep-sea sediment samples at 3144 m (11°130,900 N, 139°5505000E), Mariana Trench area, Pacific Ocean.
Data accessibility	Repository name: NCBI Data identification number: PRJNA1142708 Direct URL to data: https://www.ncbi.nlm.nih.gov/sra/PRJNA1142708 The raw sequences have been deposited in the SRA database of NCBI (PRJNA1142708). Instructions for accessing these data: The raw sequencing reads, and the transcriptome assembly can be accessed and downloaded by visiting the direct URL. The filtered sequencing reads are also available through the URL.
Related research article	None

## 1 Value of the Data

• This data includes sequencing reads and the first draft transcriptome assembly for *Mayorella marianaensis*, a naked amoeba species from deep sea.
• Among all deep-sea eukaryotic organisms like mussel, protozoa such as naked amoebas have a simple structure, and their gene expression is relatively straightforward, making their transcriptome data easier to study and interpret. This simplicity provides significant research value in fields like evolution, gene function, and environmental adaptability.
• By studying the transcriptomic data of *M. marianaensis*, we could probably uncover ancient metabolic pathways that offer clues about early biological evolution. Meanwhile, many protozoa occupy crucial positions in the evolutionary transition between prokaryotes and eukaryotes, making them ideal subjects for phylogenetic analyses and evolutionary comparisons.
• The transcriptome dataset is useful to study gene expression changes in response to extreme environment. Researchers can identify genes, proteins or molecular pathways involved in tolerance to high pressure, cold temperatures, low oxygen, and nutrient scarcity. We can gain profound insights into the biochemical and molecular strategies that enable these organisms to thrive in one of Earth's most challenging ecosystems.
• Our transcriptome data also could be integrated into various bioinformatic workflows for meta-analyses, and utilized for testing hypotheses about gene function in extreme conditions.
• This transcriptome sequences will act as essential references and valuable reservoirs for the further study of creatures in deep sea.

## 2 Background

Till now, almost 220 naked amoeba species were reported in marine and brackish-water biotopes [1]. However, only nine *Mayorella* species were reported from marine habitats [1,2], of which only one species, *Mayorella* sp., was collected from a relatively deep area (993 m) in the Mediterranean Sea [2]. In general, data on deep sea amoebae are still scarce. In present study, we firstly isolated and successfully cultivated a *Mayorella* species from deep sea in Pacific Ocean [3]. It’s a magnificent challenge for creatures to inhabit deep sea with darkness, low temperature and high hydrostatic pressure. Thus, it’s very important to figure out how those organisms adapt to harsh and extreme environment. Therefore, we conducted transcriptomic analysis of *M. marianaensis* and tried to find some certain genes relate to deep-sea environmental adaptability. Meanwhile, we provided the data generated together with the complete information, regarding methodological details and bioinformatics procedures, to make these data easily reusable by the research community.

## 3 Data Description

**Fig. 1:**
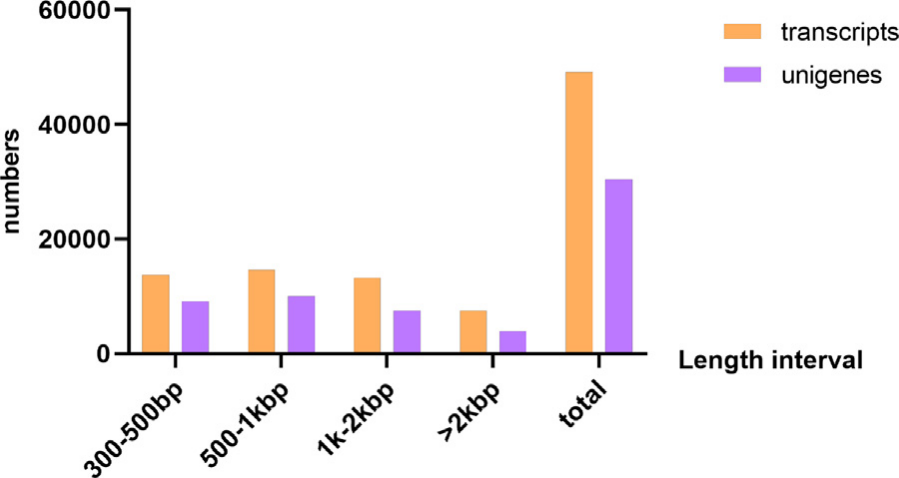
Assemblys statistics of *M. marianaensis*. Note: Length_interval: indicates the different length intervals for splicing Transcript/unigene. Number of transcripts: indicates the number of transcripts in the corresponding length interval. Number of unigenes: indicates the number of unigenes in the corresponding length interval.

**Fig. 2:**
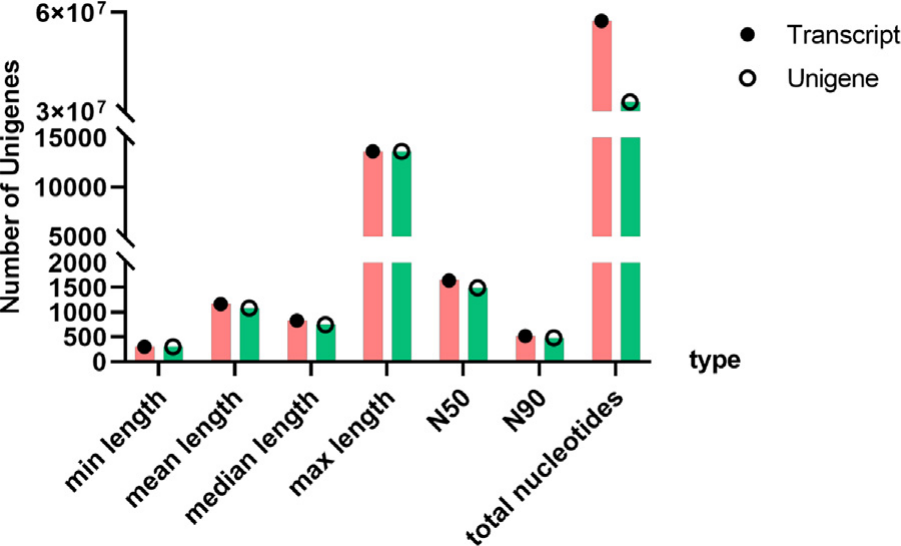
Length distribution of Transcripts and Unigenes. Note: N50: The assembled transcripts are sorted by length from longest to shortest. The length of the transcript at which the cumulative length reaches at least 50 % of the total length of all assembled transcripts is called N50. It is used to assess the assembly quality. N90: The assembled transcripts are sorted by length from longest to shortest. The length of the transcript at which the cumulative length reaches at least 90 % of the total length of all assembled transcripts is called N90. It is used to assess the assembly quality.

**Fig. 3:**
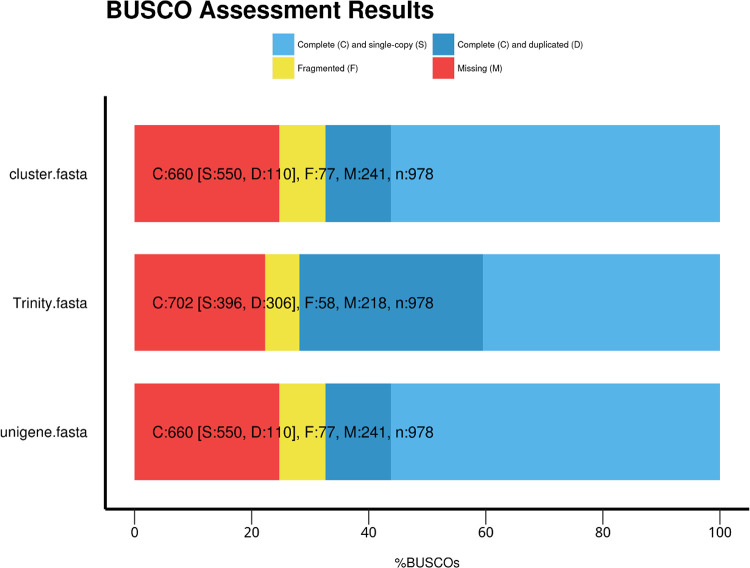
BUSCO evaluation results of spliced transcripts. Note: S:Complete Single-Copy BUSCOs. D:Complete Duplicated BUSCOs. F:Fragmented BUSCOs. M:Missing BUSCOs. n:Total BUSCO groups searched.

Transcriptomic data of *M. marianaensis* was generated using Illumina technology. 22,747,662 sequencing reads were collected totalling 2.06 Gb. A total of 21,589,280 clean reads were obtained. The Q20 percentage was 97.56 % and the GC content was 38.7 % (Table 1). The *de novo* assembly produced 57,459,544 total nucleotides for transcripts with an average of 1170 bp (Fig. 1), and N50 of 1646 bp and 32,949,799 nucleotides for unigenes with average 1083 bp and N50 of 1495 bp (Fig. 2). The N50 value is commonly used as an indicator of assembly quality, with an N50 greater than 1000 bp typically indicating a high-quality assembly. The transcriptome has completeness of 71 % (Trinity) and 67.4 % (Unigene)as assessed by BUSCO (Fig. 3).

**Table 1:** Summary of RNA-seq generated from *M. marianaensis* species.

sample	raw_reads	clean_reads	error_rate	Q20 ( %)	Q30 ( %)	GC ( %)
*M. marianaensis*	22,747,662	21,589,280	0.03	97.56	93.25	38.70

sample: sample name.

raw_reads: The number of reads before filtering.

clean_reads: The number of reads after filtering.

error_rate: The percentage of incorrect bases in the raw reads.

Q20: The percentage of bases whose quality was greater than 20 in the clean reads.

Q30: The percentage of bases whose quality was greater than 30 in the clean reads.

GC: The content of bases G and C in the clean reads.

After the spliced transcripts were obtained, gene function annotations of seven major databases (NR, NT, KO, SwissProt, PFAM, GO, KOG) were carried out to get comprehensive gene function information. A total of 1392 unigenes (4.57 %) were annotated in all databases and 22,848 unigenes were annotated in at least one Database. Among the annotated unigenes, 19,035 (62.54 %) were obtained from GO, followed by 19,035 (62.54 %) in PFAM, 16,933 (55.63 %) in NR, 14,682 (48.23 %) in SwissProt, 10,197 (33.50 %) in KO, 9106 (29.91 %) and 2153 (7.07 %) in KOG and NT, respectively (Table 2).

**Table 2:** Statistics of gene annotation success rate.

Database	Number of Unigenes	Percentage ( %)
Annotated in all Databases	1392	4.57
Annotated in at least one Database	22,848	75.06
Annotated in GO	19,035	62.54
Annotated in KO	10,197	33.50
Annotated in KOG	9106	29.91
Annotated in NR	16,933	55.63
Annotated in NT	2153	7.07
Annotated in PFAM	19,035	62.54
Annotated in SwissProt	14,682	48.23
Total Unigenes	30,436	100.00

**Fig. 4:**
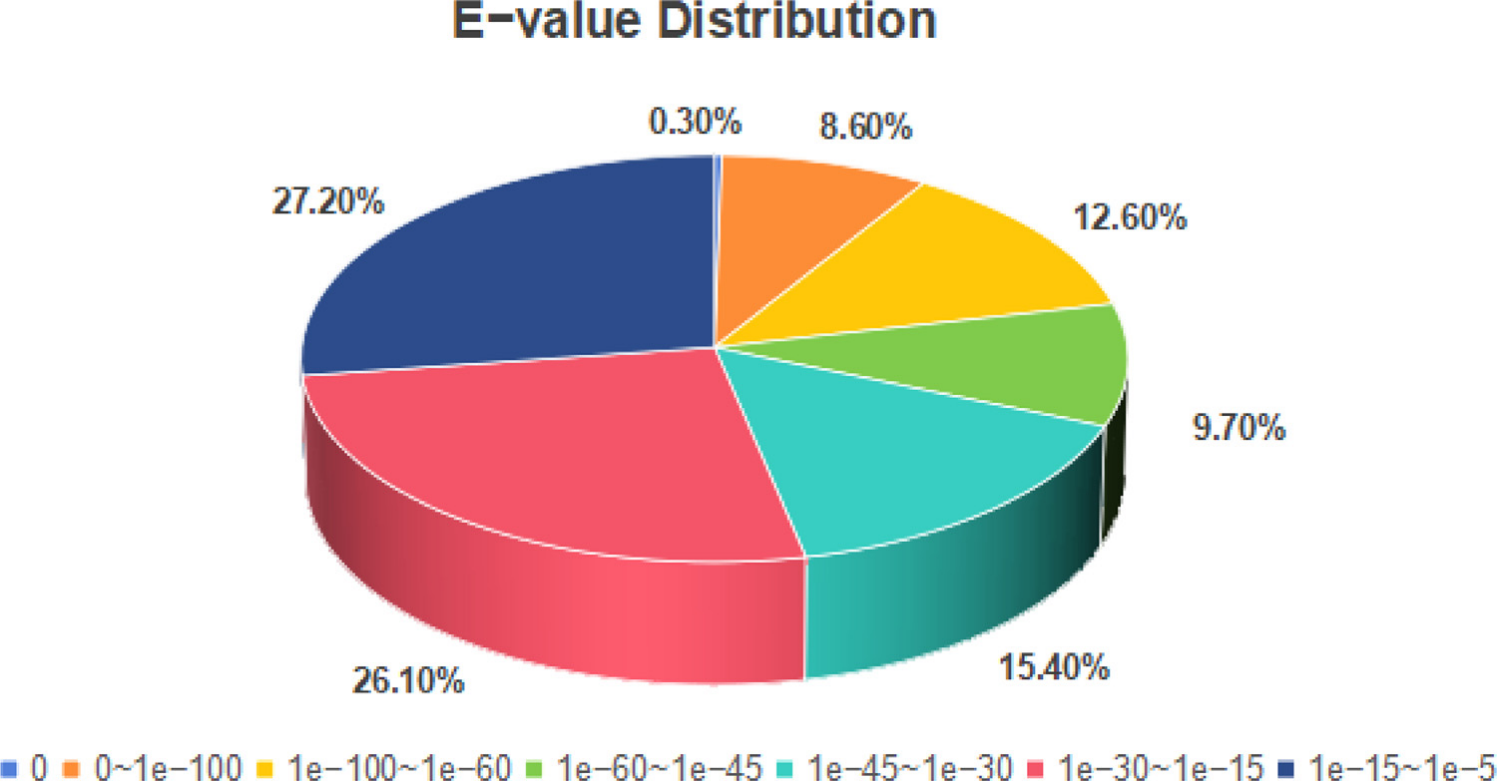
E-value Distribution Showing transcript information. Note: Evalue_Distribution is the e-value distribution diagram on the Nr library comparison.

**Fig. 5:**
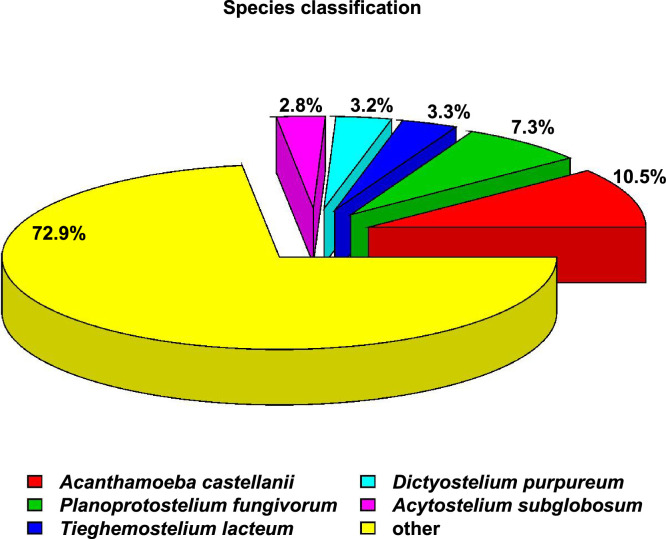
Species classification showing transcript information. Note: Species_classification is the species distribution map based on the Nr library comparison.

According to the Nr annotation results, Unigenes with an E-value of 0 account for only 0.3 % of the total, while the highest proportion, 27.2 %, falls within the E-value range of 1e-15 to 1e-5. The E-value represents the false positive rate, with smaller values indicating more reliable results. (Fig. 4). >4588 of the transcripts of *M. marianaensis* share information from five amoeba species: *Acanthamoeba castellanii, Planoprotostelium fungivorum, Dictyostelium purpureum, Tieghemostelium lacteum*, and *Acytostelium subglobosum* (Fig. 5).

**Fig. 6:**
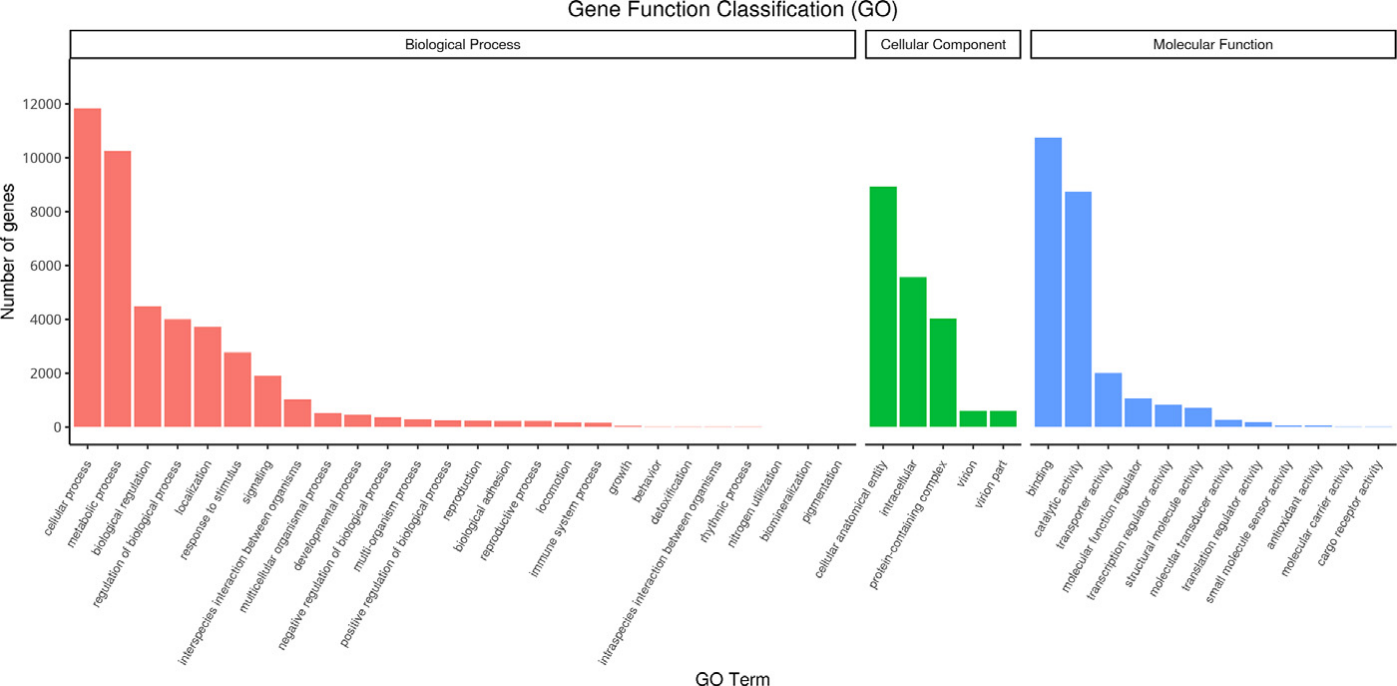
Summary of GO classification. Note: The horizontal axis in the figure is GO Term, and the vertical axis is the number of genes annotated to this GO Term.

Transcripts that mapped were annotated with GO. GO terms were categorized into three groups: cellular component, molecular function, and biological processes. The highest number of genes (11,838) was for cellular process in biological process (Fig. 6). The highest cellular component GO term consisted of 10,248 genes in relation to metabolic process whereas binding (10,749) and catalytic activity (8749) accounted for the highest in molecular function (Table S1 and Table S2). These results are consistent with the GO annotations for G*igantidas haimaensis* [4]. Cold adaptation protein (CAP), Replication factor A1 (RFA1), alanine aminotransferase, and actin were found in the *M. marianaensis* transcriptome. Although several naked amoeba species were reported in deep sea, no transcriptome data was available. In the present study, HSP (heat shock protein) and CSP (cold shock protein) families were annotated. Meanwhile, RFC1 (Replication factor C subunit) and RFA1, which help repair DNA damaged by environmental stress, were also identified in this amoeba species. All these results are consensus to the previous studies about deep-sea metazoan species, such as *G. haimaensis* and *Aldrovandia affinis* [4,5].

**Fig. 7:**
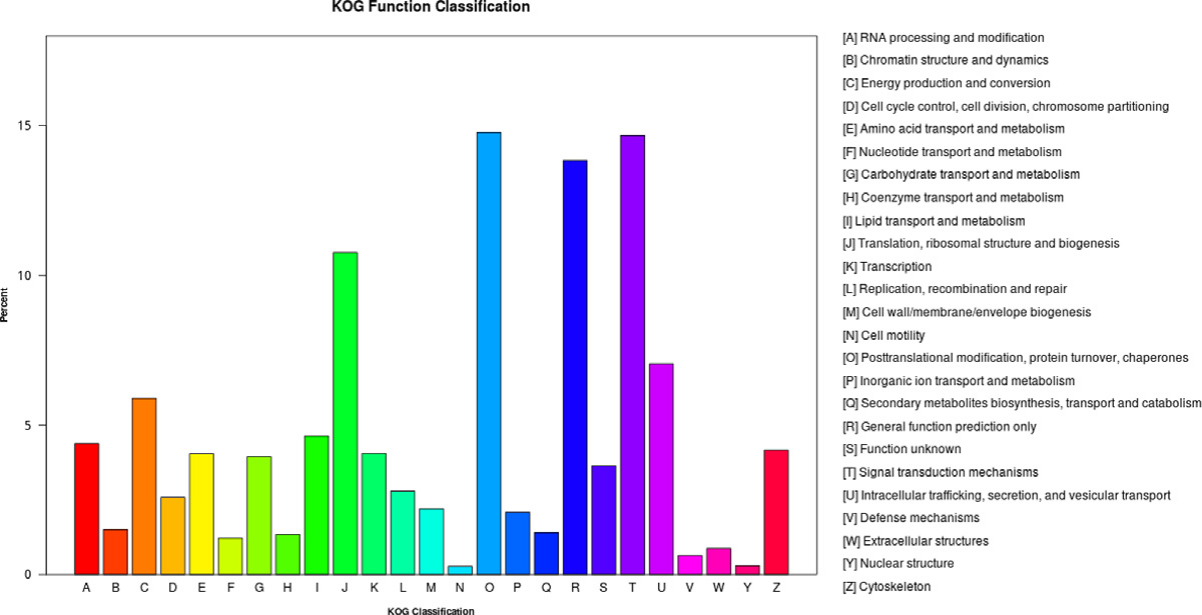
Summary of KOG classification. Note: The horizontal axis in the figure is the name of the 26 groups of KOG, and the vertical axis is the ratio of the number of genes annotated to this group to the total number of annotated genes.

A total of 9106 unigenes annotated in KOG and classified into 26 functional categories (Table S3 and Table S4). The largest function category was posttranslational modification, protein turnover, chaperones with 1345 unigenes followed by signal transduction mechanisms with 1336 unigenes (Fig. 7).

**Fig. 8:**
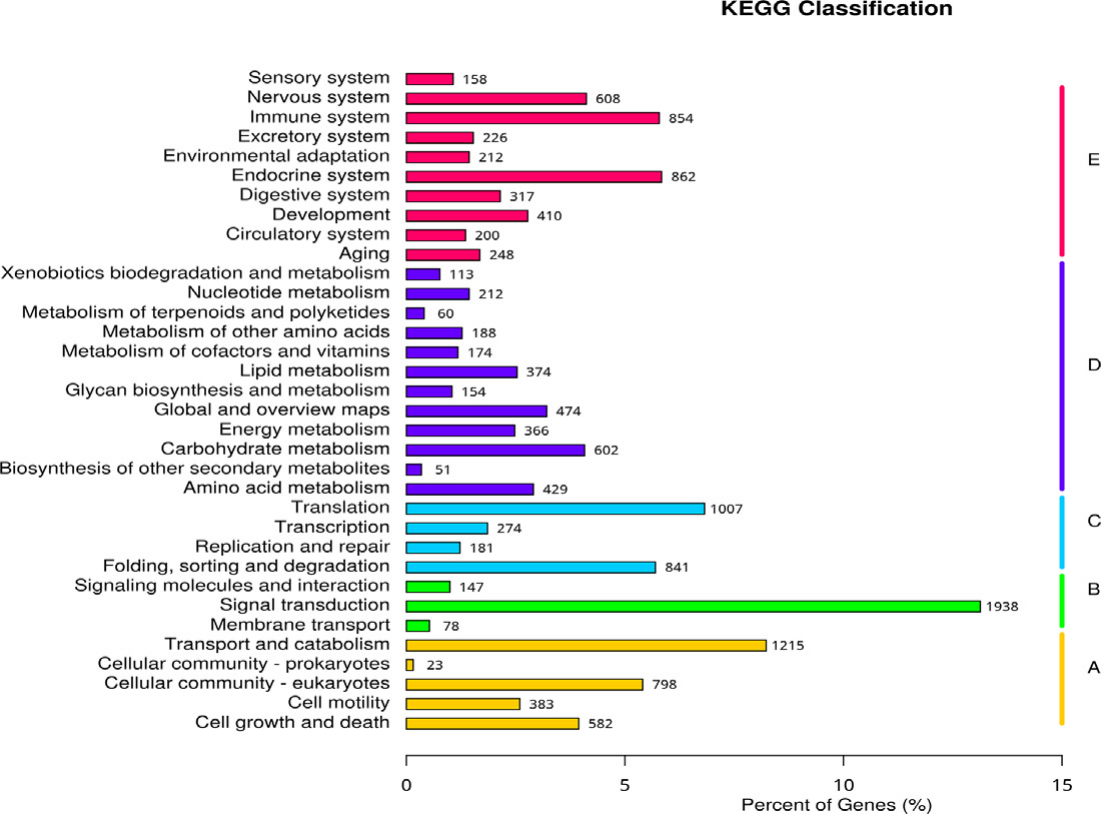
KOG annotation classification statistics. Note: The vertical axis in the figure is the name of the KEGG metabolic pathway, and the horizontal axis is the number of genes annotated to the pathway and its ratio to the total number of annotated genes. Genes are divided into 5 branches according to the KEGG metabolic pathways they participate in: Cellular Processes (A), Environmental Information Processing (B), Genetic Information Processing (C), Metabolism (D), and Organic Systems (E).

A total of 14,759 unigenes were annotated to the KEGG database (Table S5 and Table S6). Genes are divided into five branches: Cellular Processes (A), Environmental Information Processing (B), Genetic Information Processing (C), Metabolism (D), and Organic Systems (E). The top three enriched ones are signal transduction, Transport and Catabolism, and Translation (Fig. 8). Genes of signal transduction can help organisms sense and respond to extreme environmental conditions. The highest richness signal transduction related genes were also annotated in deep-sea mussel G*igantidas haimaensis* [4].

**Fig. 9:**
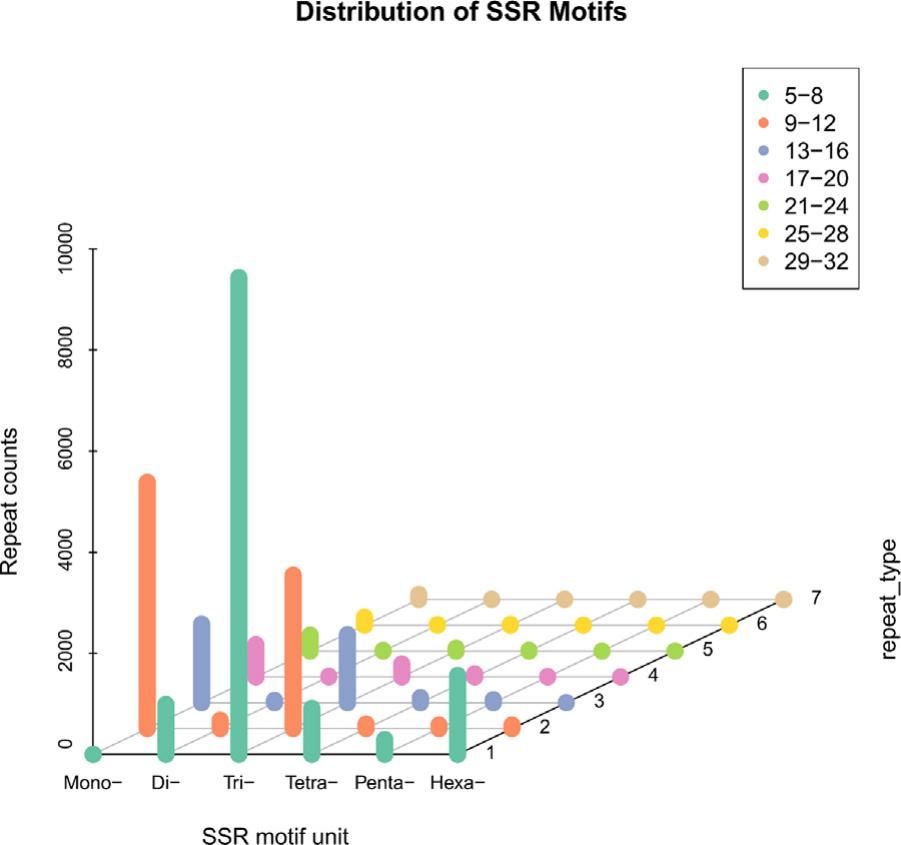
Distribution of SSR Motifs with transcript information. Note: X (SSR motif unit) coordinate is SSR type. Y (repeat_type) coordinate is the specific number of repetitions, which should correspond to the color and legend, and Z (Repeat counts) coordinate is the number of SSRs.

A total of 26,464 simple sequence repeat (SSR) loci were identified from 30,436 unigenes (Table 3), including mono-, di-, tri-, tetra-, penta-, and hexanucleotide repeat motifs (Fig. 9). Trinucleotide repeats were the most abundant type, with 14,156 loci identified (Table S7 and Table S8).

**Table 3:** Characteristics of SSRs in *M. marianensis*.

Statistical item	Number
Total number of sequences examined	30,436
Total size of examined sequences (bp)	32,949,799
Total number of identified SSRs	26,464
Number of SSR containing sequences	12,445
Number of sequences containing >1 SSR	6883
Number of SSRs present in compound formation	5284

## 4 Experimental Design, Materials and Methods

### 4.1 Cell culture and collection

A mixture of sterile artificial seawater (NaCl, 26.518 ‰; MgCl_2_, 2.447 ‰; MgSO_4_, 3.305 ‰; CaCl_2_, 1.141 ‰; KCl, 0.725 ‰; NaHCO_3_, 0.202 ‰; and NaBr, 0.083 ‰) prepared in the laboratory [6]. Single-cell cultures were performed using sterile seawater with a salinity of 30 PSU, and one sterilized rice grain was added to each petri dish and incubated continuously at room temperature of 25 °C. *M. marianaensis* cells were enriched by centrifugation and deposited in liquid nitrogen as soon as possible, and then quickly stored in an ultra-low-temperature refrigerator at −80 °C for further study.

### 4.2 RNA extraction

*Mayorella marianaensis* cells were collected, total RNA was extracted using the TRizol Universal Reagent [7]. 30–50 mg of the sample was added to 1 ml of TRizol Universal reagent and homogenized using a homogenizer. The mixture was incubated at room temperature for 5 min to fully separate the nucleic acid-protein complex. Next, 0.2 ml of chloroform was added, and the mixture was vortexed for 15 s, followed by incubation at room temperature for 3 min. The sample was then centrifuged at 12,000 rpm for 15 min, and the aqueous phase (approximately 500 µl) was carefully transferred to a new tube. An equal volume of isopropanol was added to the aqueous phase, and the mixture was incubated at room temperature for 10 min, followed by centrifugation (12,000 rpm, 4 °C, 10 min) to remove the supernatant. The RNA pellet was washed with 1 ml of 75 % ethanol and centrifuged at 10,000 rpm for 5 min at 4 °C. After removing the liquid, the tube was briefly centrifuged to remove any residual liquid. The pellet was air-dried at room temperature for 2–3 min, and RNA was dissolved in 30–100 µl of RNase-Free ddH_2_O to obtain the final RNA solution. Finally, 0.96 µg total RNA (24 ng/µl, 40.00 µl) was extracted, and the concentration and integrity were adequate for library construction and sequencing.

### 4.3 Illumina library preparation and sequencing

The concentration and integrity of the RNA were evaluated by spectrophotometry (Nanodrop) and the RNA Nano 6000 Assay Kit of the Bioanalyzer 2100 system (Agilent Technologies, CA, USA), respectively. Total RNA with RNA integrity numbers (RINs) ≥ 8.0 was used for cDNA library preparation. Total RNA samples were first enriched for polyA-tailed mRNA using Oligo(dT) magnetic beads, followed by random fragmentation of the mRNA in Fragmentation Buffer with divalent cations. Fragmented mRNA was used as a template, with random oligonucleotides as primers, to synthesize the first cDNA strand in an M-MuLV reverse transcription system. The RNA strand was then degraded by RNase H, and the second cDNA strand was synthesized using DNA polymerase I and dNTPs. The purified double-stranded cDNA underwent end repair, A-tailing, and ligation of sequencing adapters. AMP XP beads were used to select cDNA fragments of 370–420 bp, followed by PCR amplification and another round of purification. The size-selected, adaptor-ligated cDNA were treated with 3 µL USER Enzyme (NEB, USA) at 37 °C for 15 min followed by 5 min at 95 °C before PCR. Then PCR was performed with Phusion High-Fidelity DNA polymerase, Universal PCR primers and Index (X) Primer. Finally, PCR products were purified (AMPure XP system) and library quality was assessed on the Qubit2.0 Fluorometer, Agilent Bioanalyzer 2100 system and qRT-PCR. The clustering of the index-coded samples was performed on a cBot Cluster Generation System using TruSeq PE Cluster Kit v3-cBot-HS (Illumina) according to the manufacturer’s instructions. After cluster generation, the library preparations with final concentration of 400 ng were sequenced on an Illumina Novaseq 6000 platform and paired-end reads were generated.

### 4.4 Sequence quality control, transcriptome assembly and annotation

The base quality of the raw data was assessed using Trimmomatic [8], reads containing adapter or primer sequences, those with >10 % undetermined bases, and low-quality reads with >50 % bases having a Qphred score below the threshold were excluded. The remaining high-quality reads were retained for assembly. Transcript sequences were derived from paired-end reads, and assembly was conducted using Trinity v2.4.0 [9,10] which involves three modules: Inchworm, Chrysalis, and Butterfly, with the default minimum k-mer coverage set to 2, generating the final TRINITY. fasta file as described by Bolger [8]. The completeness of the assembly was evaluated using Benchmarking Universal Single-Copy Orthologs (BUSCO) version3.0.2 [11] with the metazoa_odb10.

To obtain non-redundant reads and gene-level counts for each sample, Corset version 4.6 [12] was used to hierarchically cluster the transcripts based on shared reads. Corset initially aggregated transcripts with similar sequences into preliminary clusters. It then refined these clusters by incorporating transcript expression levels and applying the H—Cluster algorithm. This process separated transcripts with notably distinct expression patterns from the original clusters and formed new clusters. As a result, each cluster was designated as a “gene” which helped reduce transcript redundancy and enhanced the detection rate of differentially expressed genes (DEGs). The Kraken tool was then used to remove contaminant bacterial, archaeal, and viral transcripts from the final assembly [13]. The longest sequence in each cluster was selected for further analysis (https://code.google.com/p/corsetproject/). For mapping, the Trinity assembly was used as the reference sequence, and reads were aligned using RSEM v1.2.15 [14] and bowtie2 with the mismatch parameter set to 0. Reads with low mapping quality (<10), unmatched reads, and reads mapping to multiple genomic regions were filtered out.

All unigenes were annotated on seven major databases (Nr, Nt, Pfam, KOG/COG, Swiss-prot, KEGG, and GO) to gather comprehensive gene function information

Unigenes were annotated to the NT, NR, KOG and SwissProt databases using NCBI blast 2.2.28+ version:2.2.28+ [15], or Diamond version: v0.8.22 [16]. GO annotation was performed using blast2go version: b2g4pipe_v2.5 [17], and Pfam was conducted using hmmscan version hmmer 3 (Table S9**)**. SSRs analysis was conducted using MISA version 1.0.

## Limitations

Not applicable.

## Ethics Statement

The authors have read and follow the ethical requirements for publication in Data in Brief and confirming that the current work does not involve human subjects, animal experiments, or any data collected from social media platforms.

## CRediT authorship contribution statement

**Wenli Guo:** Conceptualization, Investigation, Writing – original draft. **Xiaoli Lei:** Data curation. **Chen Liang:** Writing – review & editing, Supervision, Project administration. **Jianming Chen:** Supervision, Resources.

## Data Availability

NCBITranscriptome of an amoeba from deep sea (Original data)

## Appendix Supplementary materials

Image, application 1

Image, application 2

Image, application 3

Image, application 4

Image, application 5

Image, application 6

Image, application 7

Image, application 8

Image, application 9

## References

[1] Kudryavtsev A., Volkova E., Voytinsky F. (2021). A checklist of amoebozoa species from marine and brackish-water biotopes with notes on taxonomy, species concept and distribution patterns. Protistol.

[2] Hausmann K., Hülsmann N., Polianski I., Schade S., Weitere M. (2002). Composition of benthic protozoan communities along a depth transect in the eastern Mediterranean Sea. Deep Sea Res. Part Oceanogr. Res. Pap..

[3] Lei X., Chen X., Chen J., Liang C. (2023). A New *Mayorella* species isolated from the Mariana Trench Area (Pacific Ocean). Protist.

[4] Zhang H., Yao G., He M. (2022). Transcriptome analysis of gene expression profiling from the deep sea in situ to the laboratory for the cold seep mussel Gigantidas haimaensis. BMC Genomic..

[5] Lan Y., Sun J., Xu T., Chen C., Tian R., Qiu J., Qian P. (2018). *De novo* transcriptome assembly and positive selection analysis of an individual deep-sea fish. BMC Genomic..

[6] Liang C., Wang W., Dong L., Mukhtar I., Wang F., Chen J. (2021). A new *protocruzia* species (Ciliophora: protocruziida) isolated from the Mariana Trench Area. Front. Microbiol..

[7] Aumsuwan P., Khan S.I., Khan I.A., Walker L.A., Dasmahapatra A.K. (2016). Gene expression profiling and pathway analysis data in MCF-7 and MDA-MB-231 human breast cancer cell lines treated with dioscin. Data Brief.

[8] Bolger A.M., Lohse M., Usadel B. (2014). Trimmomatic: a flexible trimmer for Illumina sequence data. Bioinformatics.

[9] Haas B.J., Papanicolaou A., Yassour M., Grabherr M., Blood P.D., Bowden J. (2013). *De novo* transcript sequence reconstruction from RNA-seq using the Trinity platform for reference generation and analysis. Nat. Protoc..

[10] Grabherr M.G., Haas B.J., Yassour M., Levin J.Z., Thompson D.A., Amit I., Adiconis X., Fan L., Raychowdhury R., Zeng Q., Chen Z., Mauceli E., Hacohen N., Gnirke A., Rhind N., Di Palma F., Birren B.W., Nusbaum C., Lindblad-Toh K., Friedman N., Regev A. (2011). Full-length transcriptome assembly from RNA-seq data without a reference genome. Nat. Biotechnol..

[11] Manni M., Berkeley M.R., Seppey M., Zdobnov E.M. (2021). BUSCO: assessing genomic data quality and beyond. Curr. Protoc..

[12] Davidson N.M., Oshlack A. (2014). Corset: enabling differential gene expression analysis for *de novo* assembled transcriptomes. Genome Biol.

[13] Wood D.E., Salzberg S.L. (2014). Kraken: ultrafast metagenomic sequence classification using exact alignments. Genome Biol.

[14] Li B., Dewey C. (2011). RSEM: accurate transcript quantification from RNA-seq data with or without a reference genome. BMC Bioinf.

[15] Altschul S.F., Madden T.L., Schäffer A.A., Zhang J., Zhang Z., Miller W., Lipman D.J. (1997). Gapped BLAST and PSI-BLAST: a new generation of protein database search programs. Nucleic. Acids Res.

[16] Buchfink B., Xie C., Huson D.H. (2015). Fast and sensitive protein alignment using DIAMOND. Nat. Meth..

[17] Götz S., García-Gómez J.M., Terol J., Nagaraj S.H., Nueda M.J., Robles M., Williams T.D., Joaquín D., Coneas A., Talón M. (2008). High-throughput functional annotation and data mining with the Blast2GO suite. Nucleic Acids. Res.

