# Supplementary material for: De novo transcriptome dataset of a Mayorella species isolated from deep sea
Source: Data Brief. 2025 Jul 9;61:111864. doi: 10.1016/j.dib.2025.111864 (PMC12296473; doi:10.1016/j.dib.2025.111864)
Supplement: Supplementary file 9 [file mmc9.docx]

**Table S9**

Software versions and parameters used.

| Software | Version | Parameters |
| --- | --- | --- |
| NCBI blast | v2.2.28+ | e-value = 1e-5 |
| Diamond | v0.8.22 | e-value = 1e-5 |
| blast2go | b2g4pipe v2.5 | e-value = 1.0E-6 |
| Trinity | v2.4.0 | min_kmer_cov:3 |
| Corset | version 4.6 | default parameter |
| hmmscan | HMMER 3 | e-value = 0.01 |
| RSEM | v1.2.15 | bowtie2 mismatch 0 |
